# The immunomodulatory role of postbiotics and parabiotics: a narrative review of next-generation probiotics

**DOI:** 10.3389/fnut.2026.1966683

**Published:** 2026-10-26

**Authors:** Ülkü Demirci

**Affiliations:** Department of Nutrition and Dietetics, Faculty of Health Sciences, Istanbul Aydın University, Istanbul, Türkiye

**Keywords:** immunomodulation, intestinal barrier, next generation probiotics, parabiotics, postbiotics, Toll-like receptors

## Abstract

**Background:**

Traditional live probiotics present profound translational challenges, including manufacturing instability and systemic safety risks in immunocompromised patients. To overcome these constraints, non-viable alternatives—namely postbiotics and parabiotics—have emerged as promising biologically active components within the framework of next-generation probiotics (NGPs).

**Objective:**

This study reviews current clinical and preclinical evidence regarding the molecular mechanisms and therapeutic efficacy of postbiotics and parabiotics in modulating the host’s innate and adaptive immune responses.

**Methods:**

PubMed, Web of Science, Scopus, and the Cochrane Library were systematically searched for eligible human and animal studies published up to May 2026 in accordance with the Preferred Reporting Items for Systematic Reviews and Meta-Analyses (PRISMA) guidelines. Following rigorous screening and selection, a total of 11 studies that met the inclusion criteria were evaluated to assess the immunological outcomes associated with inanimate microbial fractions and metabolic byproducts.

**Results:**

Findings indicate that parabiotic molecular pathways primarily rely on interactions between microbe-associated molecular patterns (MAMPs) derived from the cell wall and host pattern recognition receptors (PRRs), specifically Toll-like receptor 2 (TLR-2) and nucleotide-binding oligomerization domain-like receptors 1 and 2 (NOD1/NOD2). Concurrently, postbiotics (short-chain fatty acids, exopolysaccharides, and extracellular vesicles) downregulate pro-inflammatory cascades through nuclear factor-κB (NF-κB) inhibition and upregulate regulatory T cells (Tregs) through interleukin-10 (IL-10) induction.

**Conclusion:**

By reducing bacterial translocation risks and horizontal antibiotic resistance gene transfer, while ensuring high techno-functional stability, postbiotics and parabiotics exert potent, reproducible immunomodulatory effects, establishing them as safe candidates for targeted biotherapeutic applications.

## 1 Introduction

Therapeutic manipulation of the human gastrointestinal microbiome has historically relied on the administration of traditional live probiotics, which have overwhelmingly been dominated by the genera *Lactobacillus*, *Bifidobacterium*, and *Saccharomyces* (1). While these classical strains have shown efficacy in maintaining general gut homeostasis and preventing acute gastrointestinal disturbances, their clinical and industrial applications are hindered by significant bioprocessing and physiological challenges.

Techno-functional limitations, such as high vulnerability to gastric acidity, bile salt degradation, and poor viability retention during extended industrial shelf life, necessitate expensive stabilization techniques such as microencapsulation and continuous cold-chain infrastructure (2).

More critically, a shift in clinical safety paradigms has exposed serious physiological hazards associated with the delivery of live biotherapeutics. Administering viable microorganisms carries inherent risks of opportunistic bacteremia or sepsis, systemic microbial translocation across compromised epithelial barriers, and horizontal transfer of antibiotic-resistance genes to endogenous pathogens (3). These safety liabilities strictly preclude the use of traditional live probiotics in vulnerable, critically ill, neonatal, or severely immunocompromised patient cohorts, creating a significant clinical gap.

To address these limitations, recent advancements in microbiome research have facilitated a methodological transition toward non-viable microbial alternatives, coalescing under the taxonomy of next-generation probiotics (NGPs) (4). Progress in multi-omics technologies has demonstrated that the physiological benefits of probiotics often do not depend on cell viability; instead, they are mediated by structural cell-wall components and synthesized metabolic sub-products. Consequently, utilizing inanimate microbial structures and cell-free metabolites has emerged as a safer, more stable, and highly standardizable pharmacological strategy.

The rapid expansion of the NGP literature has historically been characterized by terminological ambiguity. To establish rigorous empirical boundaries, this narrative review adheres strictly to the consensus definitions codified by the International Scientific Association for Probiotics and Prebiotics (ISAPP) (5). Parabiotics are defined as non-viable, inanimate microbial cells containing cell-wall components that can confer health benefits, generated through controlled inactivation processes (6). Postbiotics refer to preparations of inanimate microorganisms and/or their cell-free multi-component matrices, including short-chain fatty acids (SCFAs), exopolysaccharides (EPS), and extracellular vesicles (EVs) (7). This study reviews current clinical and preclinical evidence on the molecular mechanisms and therapeutic efficacy of postbiotics and parabiotics in modulating the host’s innate and adaptive immune responses.

## 2 Methodology

### 2.1 Search strategy and bibliographic databases

This narrative review was designed and conducted in strict accordance with the Preferred Reporting Items for Systematic Reviews and Meta-Analyses (PRISMA) statement (8). A comprehensive electronic search was systematically conducted across four high-impact bibliographic databases, namely PubMed/MEDLINE, Web of Science (Core Collection), Scopus, and the Cochrane Library. The search architecture utilized combinations of Medical Subject Headings (MeSH) terms and Boolean text words to maximize retrieval sensitivity and specificity. The foundational search string was structured as follows:

(“next-generation probiotics” OR “parabiotics” OR “postbiotics” OR “heat-killed probiotics” OR “non-viable microbial products”) AND (“immune system modulation” OR “immunomodulation” OR “cytokine profile” OR “Toll-like receptors” OR “intestinal epithelial barrier”) AND (“systematic review” OR “clinical trial” OR “*in vivo*”).

The temporal parameters for publication inclusion spanned from database inception up to May 2026, with language filters restricted to English-language peer-reviewed literature. Gray literature, abstract-only conference proceedings, and non-peer-reviewed preprints were systematically excluded.

### 2.2 Inclusion and exclusion criteria

To qualify for inclusion in this narrative synthesis, candidate studies were required to meet stringent eligibility criteria. The study population had to include *in vivo* animal models (murine or porcine) that exhibited induced immune dysregulation, inflammatory disorders, or metabolic syndromes, and human randomized controlled trials (RCTs) evaluating immune outcomes. For the intervention, studies needed to utilize monotherapy or well-defined combination therapy with characterized parabiotics (fully inactivated) or cell-free postbiotic preparations. Additionally, trials had to include a concurrent placebo, a negative control vehicle, or an active reference standard, such as traditional live probiotic controls. The primary outcomes required quantifiable metrics of immunomodulation, including, but not limited to, alterations in pro- and anti-inflammatory cytokine concentrations (tumor necrosis factor-α (TNF-α), interleukin-6 (IL-6), interleukin-10 (IL-10), and transforming growth factor-β (TGF-β)), expression patterns of tight junction proteins (claudin-1, occludin, and zonula occludens-1 (ZO-1)), receptor binding affinity profiles (Toll-like receptor 2/4 (TLR-2/TLR-4)), and changes in regulatory T-cell (Treg) phenotypes. Conversely, studies evaluating live probiotic formulations without distinct inanimate arms or those using undefined chemical fractions lacking microbial provenance were excluded.

### 2.3 Study selection process

The retrieved records were first exported to a reference-management file, and duplicate entries were removed before screening. Titles and abstracts were screened based on predefined Population, Intervention, Comparison, Outcome, and Study design (PICOS) criteria, followed by a full-text eligibility assessment. Studies were retained only when the intervention was clearly described as an inactivated microbial preparation, a parabiotic, a postbiotic, a microbial metabolite, an extracellular vesicle, or a cell-free microbial fraction, and when at least one immune-related outcome was reported. Disagreements during screening were resolved through discussion and, when necessary, by reassessing the studies in line with the operational definitions proposed by ISAPP and the PRISMA 2020 reporting framework (5, 8).

### 2.4 Data extraction

A standardized data-extraction form was used to collect the following variables from each eligible study: author and year, country or setting, study design, population or model, sample size, microbial strain or postbiotic/parabiotic source, inactivation or preparation method, dose and duration, comparator group, biological matrix, primary immune outcomes, molecular signaling pathways, intestinal barrier markers, microbiota or metabolomic outcomes, and main findings. Particular attention was paid to cytokine profiles (TNF-α, interleukin-1β (IL-1β), IL-6, IL-10, interleukin-12 (IL-12), interleukin-17 (IL-17), TGF-β, and interferon-γ (IFN-γ)), receptor-level mechanisms (TLR2, TLR4, TLR9, nucleotide-binding oligomerization domain-like receptors 1 and 2 (NOD1/NOD2), and G-protein-coupled receptors (GPCRs)), transcriptional regulators (nuclear factor-κB (NF-κB), mitogen-activated protein kinase (MAPK), histone deacetylase (HDAC), and peroxisome proliferator-activated receptor-γ (PPAR-γ)), and cellular immune responses such as dendritic cell maturation, macrophage activation, secretory immunoglobulin A (sIgA) secretion, and Treg differentiation (9–12).

### 2.5 Quality assessment and risk of bias

Methodological quality was assessed according to study design. For randomized human trials, the Cochrane risk-of-bias 2 (RoB 2) framework was considered to evaluate randomization, deviations from intended interventions, missing outcome data, outcome measurement, and selective reporting domains (13). For animal experiments, the Systematic Review Centre for Laboratory Animal Experimentation (SYRCLE) risk-of-bias tool was considered for evaluating sequence generation, baseline comparability, allocation concealment, random housing, blinding, incomplete outcome data, and selective reporting (14). Mechanistic *in vitro* studies were evaluated narratively by focusing on strain characterization, reproducibility of inactivation, analytical validation of metabolites or vesicles, dose justification, the use of appropriate inflammatory controls, and reporting of pathway-level endpoints. Due to heterogeneity in preparations, populations, and outcomes, the evidence was synthesized qualitatively rather than being statistically pooled.

### 2.6 PRISMA flow diagram

Figure 1 presents the PRISMA-based study-selection structure. Exact numerical values should be obtained from the final database export logs to avoid introducing unverifiable counts; however, the diagram clarifies the required reporting sequence from identification to final inclusion (8).

**Figure 1:**
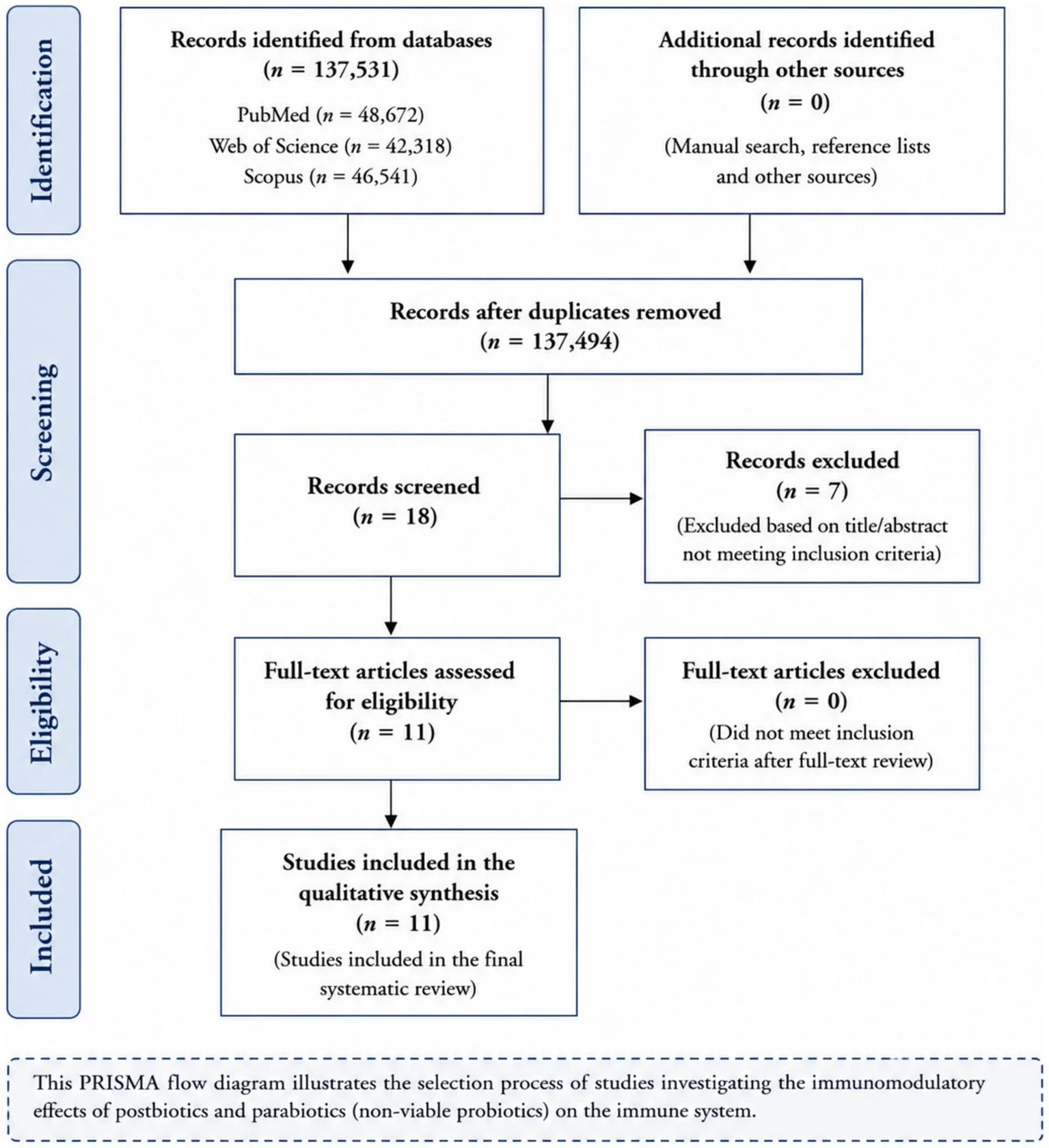
PRISMA flow diagram.

### 2.7 Evidence overview strategy

Due to substantial heterogeneity across study populations, microbial strains, inactivation methods, outcome measures, and experimental designs, a quantitative meta-analysis was considered inappropriate. Therefore, findings were synthesized narratively. Evidence was grouped according to study type, intervention characteristics, molecular mechanisms, immune outcomes, and clinical applications. Particular emphasis was placed on identifying recurring immunomodulatory pathways, including Toll-like receptor (TLR)-mediated signaling, NF-κB and MAPK modulation, SCFA-mediated epithelial barrier regulation, and Treg-associated immune responses. The overall strength of evidence was interpreted based on consistency of findings across human, animal, and mechanistic studies.

## 3 Results

### 3.1 Cellular and molecular mechanisms of immunomodulation

#### 3.1.1 Receptor binding dynamics: parabiotic-host cell crosstalk

The mechanism driving parabiotic efficacy depends on the direct physical interaction between non-viable structural elements and the host’s innate immune architecture. Parabiotics function as concentrated carriers of microbe-associated molecular patterns (MAMPs). The primary sensors for these components are host pattern recognition receptors (PRRs), predominantly Toll-like receptor 2 (TLR-2) and nucleotide-binding oligomerization domain-like receptors (NOD1 and NOD2), which are highly expressed on the surfaces of intestinal epithelial cells (IECs), dendritic cells (DCs), and mucosal macrophages. Lipoteichoic acid (LTA) in the cell walls of heat-killed *Lactobacilli* exhibits high binding affinity for the extracellular domain of TLR-2, while peptidoglycan (PGN) fragments are recognized by both TLR-2 and the intracellular pattern-recognition receptors NOD1/NOD2 (10). Notably, LTA does not reliably activate TLR-4 signaling; rather, it may form an inactive LTA-TLR4/myeloid differentiation factor 2 (MD-2) complex dimer that antagonizes TLR-4-mediated responses (15). TLR-2 and NOD1/NOD2 ligation subsequently triggers an intracellular signaling cascade governed by the myeloid differentiation primary response 88 (MyD88) adapter protein (12).

#### 3.1.2 Intestinal epithelial barrier reinforcement by postbiotics

Postbiotic metabolites, particularly short-chain fatty acids (SCFAs), upregulate tight junction protein transcription. SCFAs, particularly butyrate, act as primary signaling ligands for host G-protein-coupled receptors (specifically GPR41 and GPR43) and function as potent histone deacetylase (HDAC) inhibitors (16). At the transcriptomic level, butyrate-mediated HDAC inhibition accelerates the expression of genes encoding critical apical junctional complexes: claudin-1, occludin, and zonula occludens-1 (ZO-1) (17, 18).

#### 3.1.3 Integrated molecular signaling pathways

As detailed in Table 1, current evidence indicates that postbiotic and parabiotic fractions engage distinct cellular receptors. Parabiotic MAMPs primarily engage TLR2 and NOD1/NOD2, activating MyD88-dependent signaling (10). Postbiotic fractions, particularly SCFAs such as butyrate, interact with GPR41/GPR43 and modulate nuclear signaling by inhibiting HDAC activity—thereby increasing histone acetylation—while activating PPAR-γ, collectively promoting anti-inflammatory gene expression and epithelial barrier integrity (11, 16).

**Table 1:** Expanded comparison of live probiotics, parabiotics, and postbiotics with molecular signaling pathways.

Parameter	Live probiotics	Parabiotics	Postbiotics
Primary physical entity	Viable, metabolically active cell (1)	Dead cell carcass/structural remnants (6)	Soluble metabolites/micro-vesicles (7)
Receptor selectivity	Variable, colonization-dependent (1)	High affinity for TLR-2 and NOD2 (10, 15)	Diverse; binds GPR41/43 and PPAR-γ (11, 16)
Sepsis and bacteremia risk	Present in vulnerable cohorts (3)	Completely eliminated (2, 4)	Completely eliminated (2, 4)
Antibiotic gene transfer	High risk (plasmid-mediated) (3)	Abolished (2, 4)	Abolished (2, 4)
Gastrointestinal survival	High loss via gastric acid/bile (2)	Independent of survival kinetics (19)	Exempt from gastrointestinal transit loss (19, 20)
Manufacturing stability	Low; high moisture sensitivity (2)	High thermal and physical stability (19, 35)	Excellent matrix integration capacity (19, 20)
Dominant molecular signals/pathways	Colonization-dependent MAMP and metabolite signals; strain-specific epithelial crosstalk (33)	TLR2, NOD1/NOD2, MyD88 signaling, dendritic-cell maturation, and sIgA response (10, 12)	GPR41/GPR43 activation, HDAC inhibition, PPAR-γ regulation, NF-κB/MAPK modulation, and Treg/IL-10 axis (11, 16)
Key immune cells affected	Dendritic cells, macrophages, and epithelial cells; effect depends on viable strain persistence (33)	Dendritic cells, macrophages, natural killer cells, and epithelial PRRs through preserved cell-wall ligands (10, 12)	Epithelial cells, Tregs, macrophages, and mucosal B-cell/sIgA responses through metabolites, EPS, and EVs (25, 28)
Barrier and metabolomic markers	Variable SCFA production; requires survival and metabolic activity (16)	Can preserve MAMP-driven epithelial signaling but does not require colonization (9, 10)	Butyrate, acetate, EPS, peptides, and EV cargo promote claudin-1, occludin, and ZO-1 expression (16, 17)
Standardization concern	Viability, storage, and strain drift complicate dose equivalence (2)	Inactivation method must preserve PGN/LTA/S-layer structures (35)	Metabolite fingerprint, EV size/cargo, and batch-to-batch composition require omics validation (34, 36)

TLR, Toll-like receptor; NOD, nucleotide-binding oligomerization domain; GPR, G-protein-coupled receptor; PPAR-γ, peroxisome proliferator-activated receptor-γ; MAMP, microbe-associated molecular pattern; MyD88, myeloid differentiation primary response 88; sIgA, secretory immunoglobulin A; HDAC, histone deacetylase; NF-κB, nuclear factor-κB; MAPK, mitogen-activated protein kinase; Treg, regulatory T cell; IL-10, interleukin-10; PRR, pattern recognition receptor; EPS, exopolysaccharides; EV, extracellular vesicle; SCFA, short-chain fatty acid; ZO-1, zonula occludens-1; PGN, peptidoglycan; LTA, lipoteichoic acid.

*Molecular pathway diagram:* The interaction between specific postbiotic and parabiotic fractions (e.g., MAMPs, EPS, and EVs) and host receptors (e.g., TLRs and NODs) generally results in the activation of these receptors. This activation leads to MyD88/NF-κB/MAPK modulation, driving cytokine rebalancing (lower TNF-alpha, IL-6, and IL-17; higher IL-10 and TGF-betaβ), which subsequently promotes Treg expansion, sIgA response, epithelial barrier restoration, and immune homeostasis (9, 11, 12).

### 3.2 Technological and industrial superiority

In contrast, parabiotics and postbiotics are physically stable structures. As they do not require replication, these non-viable agents are highly resistant to thermal stress and acidic microenvironments (19). They integrate seamlessly into diverse matrices—including high-water-activity functional beverages, baked consumer goods, and ambient-temperature blister packs—without losing immunomodulatory potency. This exceptional shelf-life stability simplifies standardized dosing protocols and ensures uniform, repeatable pharmacological delivery.

Scientifically studied applications include parabiotic-fortified breads and functional flatbreads incorporating postbiotic exopolysaccharides, which successfully maintain their immunomodulatory properties and structural integrity post-baking without compromising their sensory attributes (19, 20).

### 3.3 Therapeutic applications and clinical outcomes

#### 3.3.1 Gastrointestinal disorders (inflammatory bowel disease and irritable bowel syndrome)

Extraction of clinical trial registries reveals robust therapeutic profiles for postbiotics and parabiotics in treating inflammatory bowel disease (IBD, including Crohn’s disease and ulcerative colitis) and irritable bowel syndrome (IBS) (21). In clinical trials evaluating dextran sulfate sodium (DSS)-induced colitis models, the oral administration of heat-killed *Lactobacillus plantarum* or *Akkermansia muciniphila* derivatives significantly reduced mucosal ulceration indices.

This protective effect is achieved by restoring intestinal alkaline phosphatase activity and decreasing myeloperoxidase (MPO) accumulation, leading to lower levels of mucosal neutrophil infiltration.

#### 3.3.2 Allergic pathologies and atopic dermatitis

The therapeutic efficacy of postbiotics extends beyond the gastrointestinal tract, modulating systemic allergic conditions through the gut–skin and gut–lung axes (22). In pediatric randomized controlled trials (RCTs), infants suffering from moderate-to-severe atopic dermatitis who received heat-killed *Bifidobacterium breve* showed significant reductions in their SCORing Atopic Dermatitis (SCORAD) indices.

Mechanistically, these non-viable agents restore the physiological T-helper 1/T-helper 2 (Th1/Th2) equilibrium (23). By shifting immune polarization away from Th2-driven immunoglobulin E (IgE) hyper-secretion and mast cell degranulation, they offer a safe, non-steroidal prophylactic strategy for pediatric allergy management.

Hyper-reactive allergen exposure, characterized by a Th2 bias, triggers IgE secretion and subsequently leads to an atopic flare. However, parabiotics or postbiotics intervene in this cascade by promoting the development of tolerogenic dendritic cells (DCs). These tolerogenic DCs stimulate the expansion of regulatory T cells (Tregs), effectively restoring Th1/Th2 homeostasis. Ultimately, this restored immunological balance acts to suppress and rebalance the initial hyper-reactive pathway, thereby reducing IgE secretion and mitigating atopic flares.

Furthermore, the anti-allergic action of parabiotics and postbiotics is not based solely on restoring Th1/Th2 homeostasis; preclinical models show that it also involves the active inhibition of the pro-inflammatory TLR4/NF-κB signaling pathway at epidermal and mucosal interfaces (22, 24).

#### 3.3.3 Mucosal defense against respiratory tract infections

By enhancing mucosal immunity at distal epithelial sites, postbiotics and parabiotics provide robust protection against respiratory pathogens. Systematic clinical analysis indicates that daily consumption of postbiotic supernatants upregulates the synthesis of secretory immunoglobulin A (sIgA) in the nasopharyngeal and bronchial mucosa (25).

This elevated secretion of sIgA prevents viral attachment and entry, significantly reducing the duration and symptom severity of upper respiratory tract infections (URTIs) in both pediatric and geriatric populations.

In addition to sIgA secretion, secondary clinical reports suggest that extracellular vesicles from postbiotic fractions can competitively bind viral entry receptors on respiratory epithelial cells, providing an adjunctive barrier to combat mucosal diseases (24).

### 3.4 Overview of included studies

An overview of the included literature highlights distinct pathways through which NGPs modulate immunity (Table 2). Teame et al. demonstrated that Lactobacillus-derived parabiotic and postbiotic components regulate the immune system via TLR2, TLR4, TLR9, and NOD1/NOD2 receptors (10). Specifically, peptidoglycans suppressed IL-12 and enhanced IL-10 production, while exopolysaccharides reduced IL-6, interleukin-8 (IL-8), and TNF-α (10). Gargari et al. observed that the administration of a high-bifidobacterial formulation in healthy adults significantly increased lactate levels and the acetate/butyrate ratio, linking gut microbiota modulation to immune regulation via the intestinal mucosa (26). Similarly, Hillman et al. used comparative genomics to show that Roseburia species produce immunomodulatory butyrate alongside biologically active B vitamins (27).

**Table 2:** Summary of research findings, methodologies, and anatomical locations of action.

Author and year	Participants/model	Evaluation methods	Main findings and specific location/tissue
Teame et al. (2020) (10)	*Lactobacillus*-derived parabiotics and postbiotics	Analysis of microbial components and immunological mechanisms	Location/tissue: Intestinal mucosa and systemic immune cells (dendritic cells, Tregs). Findings: Parabiotics and postbiotics regulated immune responses through TLRs, NOD receptors, and DC-SIGN pathways; enhanced anti-inflammatory cytokines (IL-10); and supported Treg differentiation.
Gargari et al. (2020) (26)	19 healthy adults	16S ribosomal RNA (rRNA) sequencing and liquid chromatography–mass spectrometry (LC–MS) metabolite analysis	Location/tissue: Gastrointestinal tract (gut lumen) and feces. Findings: Increased fecal Bifidobacterium abundance, lactate levels, and acetate/butyrate ratio and suggested immunomodulatory potential through gut microbiota modulation.
Hillman et al. (2020) (27)	11 *Roseburia* genomes	Comparative genomics and metabolic pathway analysis	Location/tissue: Colonic epithelium and gut microbiome. Findings: Roseburia species produced butyrate with immunomodulatory properties and contributed to immune regulation through bioactive metabolites.
Nataraj et al. (2020) (9)	Postbiotic and parabiotic compounds	Literature-based mechanistic evaluation	Location/tissue: Intestinal epithelial barrier and mucosal immune system. Findings: Postbiotics and parabiotics reduced inflammation, strengthened intestinal barrier integrity, and regulated immune responses through MAMP–PRR interactions.
Yeşilyurt et al. (2021) (28)	Probiotics, parabiotics, and postbiotics	Evaluation of immune-related mechanisms	Location/tissue: Intestinal mucosa and systemic circulation (macrophages, T cells). Findings: Postbiotics increased IL-10 and TGF-β production, promoted Treg activation, enhanced macrophage activity, and supported antimicrobial defense.
Jastrząb et al. (2021) (12)	Postbiotic-derived compounds	Molecular and cellular pathway analyses	Location/tissue: Intestinal epithelial cells and mucosal immune cells. Findings: Postbiotics modulated NF-κB and MAPK pathways, regulated cytokine production, and contributed to immune homeostasis.
Li et al. (2023) (29)	106 healthy adults	Analysis of CRP, IFN-γ, IL-10, sIgA, and gut microbiota	Location/tissue: Blood plasma (systemic circulation) and gut mucosa (sIgA). Findings: Synbiotic supplementation reduced CRP and IFN-γ levels, increased IL-10 and sIgA, and promoted beneficial gut bacteria associated with anti-inflammatory responses.
Gouvarchinghaleh et al. (2023) (30)	60 hospitalized COVID-19 patients	Inflammatory cytokines and biochemical markers	Location/tissue: Blood serum (systemic circulation). Findings: Functional food intervention reduced IL-1β, IL-6, IL-17, and TNF-α and increased IL-10, indicating significant immunomodulatory and anti-inflammatory effects.
Guo et al. (2024) (31)	69 adults with chronic diarrhea	Clinical symptom assessment, microbiota, and metabolomic analyses	Location/tissue: Gastrointestinal tract (colon) and fecal microbiome. Findings: Improved diarrhea symptoms, increased *Faecalibacterium prausnitzii* and butyrate levels, and supported immune regulation through microbiota-mediated metabolic changes.
Beteri et al. (2024) (18)	53 adults with prediabetes	Analysis of glycated hemoglobin (HbA1c), fasting glucose, gut microbiota, and LBP	Location/tissue: Blood plasma (systemic circulation) and intestinal epithelial barrier. Findings: Reduced HbA1c and LBP levels, improved gut microbial diversity, strengthened intestinal barrier function, and enhanced immune tolerance.
Bhoir et al. (2025) (20)	Balb/c mice	Nutritional, antioxidant, hematological, and microbiota analyses	Location/tissue: Blood (systemic circulation) and gastrointestinal tract (gut microbiome). Findings: Improved white blood cell, lymphocyte, and monocyte counts, promoted beneficial gut bacteria, and supported immune regulation through SCFA production.

Treg, regulatory T cell; TLR, Toll-like receptor; NOD, nucleotide-binding oligomerization domain; IL, interleukin; rRNA, ribosomal RNA; LC–MS, liquid chromatography–mass spectrometry; MAMP, microbe-associated molecular pattern; PRR, pattern recognition receptor; TGF-β, transforming growth factor-β; NF-κB, nuclear factor-κB; MAPK, mitogen-activated protein kinase; CRP, C-reactive protein; IFN-γ, interferon-γ; sIgA, secretory immunoglobulin A; COVID-19, coronavirus disease 2019; TNF-α, tumor necrosis factor-α; HbA1c, glycated hemoglobin; LBP, lipopolysaccharide-binding protein; SCFA, short-chain fatty acid; DC-SIGN, dendritic cell-specific intercellular adhesion molecule-3-grabbing non-integrin.

Further mechanistic insights by Yeşilyurt et al. showed that postbiotics increase IL-10 and TGF-β production, promote Treg activation, and support antimicrobial defense (28). Jastrząb et al. confirmed that postbiotics modulate NF-κB and MAPK signaling pathways to control inflammation while preserving intestinal barrier integrity (12).

In clinical settings, Li et al. showed that synbiotic supplementation in healthy adults significantly reduced plasma C-reactive protein (CRP) and IFN-γ, while increasing IL-10 and fecal sIgA (29). Regarding functional food interventions, Gouvarchinghaleh et al. reported significant reductions in pro-inflammatory cytokines (IL-1β, IL-6, IL-17, and TNF-α) in coronavirus disease 2019 (COVID-19) patients (30). This highlights the indirect postbiotic mechanism whereby bioactive substances (fibers and polyphenols) stimulate the endogenous microbiota to produce immunomodulatory SCFAs (30). Guo et al. showed that postbiotics (Probio-Eco^®^) improved chronic diarrhea symptoms while increasing butyric acid levels and the abundance of *Faecalibacterium prausnitzii* (31). Beteri et al. noted that *Bifidobacterium breve*-derived postbiotic exopolysaccharides reduced lipopolysaccharide-binding protein (LBP) levels, strengthening intestinal barrier function (18). Finally, Bhoir et al. confirmed using a Balb/c model that a protein-rich functional food premix based on millets, pulses, and oil seeds promoted the growth of *Bifidobacterium* species and stimulated SCFA production, regulating immune responses without increasing oxidative stress (20).

## 4 Discussion

This narrative review outlines a critical paradigm shift in contemporary microbiome therapeutics: the methodological and clinical transition from viability-dependent live probiotics to the highly standardized, inanimate matrices of postbiotics and parabiotics. The reviewed evidence consistently demonstrates that the immunomodulatory efficacy of next-generation probiotics (NGPs) is largely independent of microbial viability (4, 5, 7). Instead, it is strictly governed by the molecular interactions between preserved structural components [microbe-associated molecular patterns (MAMPs)] or secreted cell-free metabolites and the host’s innate immune receptors [pattern recognition receptors (PRRs)] (9, 11). Unlike traditional live probiotics, whose clinical outcomes are frequently confounded by inter-individual colonization resistance, gastric acid degradation, and the inherent risk of opportunistic bacteremia in immunocompromised cohorts (2, 3), postbiotics and parabiotics provide highly predictable pharmacological kinetics and a strong assurance against microbial translocation (19).

The first pathway involves the direct engagement of host innate immunity by parabiotic structural elements. Recent 2026 state-of-the-art literature emphasizes that TLR signaling is central to maintaining innate immunity and modulating inflammation (32). The ligation of these receptors triggers an intracellular signaling cascade governed primarily by the MyD88 adapter protein. As our data show, this parabiotic-driven TLR activation does not induce a destructive cytokine storm; instead, it tightly regulates the nuclear translocation of NF-κB and MAPK pathways to prevent excessive inflammatory responses (11, 28). This controlled activation averts hyper-inflammation while sustaining a critical baseline of immune vigilance. Ultimately, this modulates dendritic cell maturation, enabling them to present antigens to lymphocytes and secrete specific cytokines (TGF-β, IL-10), which trigger mucosal B cells to differentiate into immunoglobulin A (IgA)-producing plasma cells and enhance the antiviral secretory IgA (sIgA) response (10, 12).

The second dominant pathway centers on the profound immunometabolic effects of postbiotic fractions. By physically reinforcing apical junctional complexes in the intestinal epithelium through HDAC inhibition, postbiotics successfully prevent the passive paracellular translocation of luminal lipopolysaccharides (LPS). This mechanism effectively arrests the initial trigger for systemic metabolic endotoxemia and chronic tissue inflammation (17, 33). Furthermore, advanced 2026 multi-omics technologies highlight that the exact metabolomic fingerprint of postbiotics—including extracellular vesicles—can actively regulate specific immunomodulatory networks, such as EV-associated microRNAs and bacterial nucleic acids that modulate macrophage NF-κB target-gene expression and NOD-, LRR- and pyrin domain-containing protein 3 (NLRP3) inflammasome activity, and can even competitively bind to viral entry receptors on respiratory epithelial cells (24, 34).

Furthermore, our overview of adaptive immune responses underscores the capability of NGPs to correct imbalances in T-cell polarization. The literature consistently reports that specific postbiotic and parabiotic interventions downregulate hyper-secretion of pro-inflammatory cytokines, including TNF-α, IL-6, and IL-12, from mucosal macrophages and activated dendritic cells (12, 30). Concurrently, they facilitate the expansion of CD4 + CD25 + Foxp3 + regulatory T cells (Tregs) in the mesenteric lymph nodes, predominantly via the induction of IL-10 and TGF-β (10, 28). This immunoregulatory capacity is clinically relevant, as reflected in reduced SCORAD indices among infants with atopic dermatitis and attenuated mucosal ulceration in IBD models (21, 23). In atopic pathologies, such as atopic dermatitis and allergic rhinitis, this mechanism restores Th1/Th2 equilibrium by suppressing Th2-driven IgE hyper-secretion and subsequent mast cell degranulation (22, 23). Similarly, in chronic gastrointestinal disorders such as inflammatory bowel disease (IBD) and irritable bowel syndrome (IBS), the SCFA–Treg axis effectively mitigates excessive neutrophilic infiltration and mucosal ulceration, confirming that these inanimate matrices are not merely supportive nutritional supplements but potent targeted biotherapeutics (21, 31).

Live probiotics require stringent cold-chain logistics, multi-layer microencapsulation, and strict moisture control to maintain viable colony-forming units (CFUs) until the expiration date (2). In stark contrast, postbiotics and parabiotics offer exceptional physical and thermal stability (6, 19). The reviewed clinical evidence confirms that integrating them into complex matrices—from highly processed functional flatbreads and pre-diabetic dietary supplements to high-water-activity clinical formulations—does not compromise their ability to lower inflammatory biomarkers (e.g., C-reactive protein, lipopolysaccharide-binding protein) or optimize glucose metabolism (18, 20, 29). Moreover, these cell-free and inactivated matrices eliminate the risk of horizontal transmission of antibiotic-resistance genes (via plasmid transfer) to endogenous gut pathogens, establishing an unmatched safety profile for highly vulnerable populations, including premature neonates and patients in intensive care units (3, 11).

Despite the clear qualitative direction of the evidence, translating these findings into commercial biotherapeutics is complicated by methodological limitations and high inter-study heterogeneity (8, 26, 27). The diverse physical inactivation treatments (e.g., thermal tyndallization versus high-pressure processing) significantly alter the spatial conformation of surface immunogens, thereby modifying their PRR binding affinity and generating variable clinical efficacies (35). Furthermore, variations in fermentation substrates and extraction protocols yield highly divergent postbiotic metabolomic fingerprints (34, 36). This variability strictly hinders current efforts to establish absolute dose-efficacy equivalency and universal dose standardization for clinical practice.

Future translational research must prioritize two distinct avenues: first, the execution of large-scale, long-term, multicenter randomized controlled trials (RCTs) with standardized reporting protocols to validate optimal dosing strategies; and second, the integration of advanced multi-omics technologies (genomics, transcriptomics, and metabolomics) to characterize and establish universal industrial standards for postbiotic matrices (34). Only rigorous standardization can definitively integrate these next-generation biotherapeutics into precision clinical gastroenterology and immunology.

Similarly, the exact composition of postbiotic matrices is highly complex and challenging to standardize. Variations in fermentation substrates, ambient pH, processing temperatures, and subsequent extraction protocols yield highly divergent metabolomic fingerprints (34, 36). Consequently, asserting absolute dose-efficacy equivalency across different commercially available NGP preparations remains scientifically premature.

## 5 Conclusion

Parabiotics and postbiotics—functioning through the presentation of preserved microbe-associated molecular patterns (MAMPs) and the delivery of cell-free bioactive metabolites (such as SCFAs and exopolysaccharides)—effectively engage host innate immune receptors (TLRs, NODs) and modulate key intracellular cascades (NF-κB, MAPK, and HDAC inhibition). These precise molecular interactions successfully promote regulatory T-cell (Treg) expansion, reinforce intestinal epithelial barrier integrity, and restore Th1/Th2 homeostasis. By eliminating the physiological hazards of opportunistic bacteremia and horizontal antibiotic resistance gene transfer, while offering unparalleled thermal and industrial stability, next-generation probiotics overcome the critical translational barriers of traditional live strains. Consequently, postbiotics and parabiotics emerge not merely as safer alternatives but as highly potent, standardizable biotherapeutics with profound clinical potential in the management of gastrointestinal, allergic, and chronic inflammatory conditions. Several limitations should be considered when interpreting these findings, notably substantial methodological heterogeneity among the included studies, differences in microbial inactivation protocols, and a lack of large-scale human clinical trials focused on specific postbiotic subclasses. Future research must prioritize robust multi-omics validation, rigorous standardization of dosing regimens, and extensive randomized controlled trials to firmly establish dose–response relationships in clinical practice.
